# Draft Genomic Sequences of Bacillus amyloliquefaciens CBMAI 1301 and Bacillus subtilis CBMAI 1302, Strains with Potential for the Biocontrol of Phytopathogens

**DOI:** 10.1128/MRA.00321-21

**Published:** 2021-08-19

**Authors:** Isabella Takahashi Kitano, Iron Amoreli de Figueiredo Ribeiro, Vitor Lima Coelho, Carlos Alberto Xavier Gonçalves, Giulia Naranjo Aranha, Liza Fernandes Moutinho, Aline Silva Romão-Dumaresq, Eduardo Roberto de Almeida Bernardo

**Affiliations:** a Research and Development (R&D) Department, Agrivalle Brasil Industria e Comércio de Produtos Agrícolas Ltda, Salto, São Paulo, Brazil; b SENAI Innovation Institute for Biosynthetics and Fibers, SENAI CETIQT, Rio de Janeiro, Rio de Janeiro, Brazil; University of Arizona

## Abstract

Here, we report the draft genomic sequences of Bacillus amyloliquefaciens strain CBMAI 1301, isolated from soybean seeds, and Bacillus subtilis strain CBMAI 1302, isolated from soil. These strains have potential applications for the biological control of phytopathogens, and the sequencing of these two genomes could greatly benefit soybean cultivation.

## ANNOUNCEMENT

*Bacillus* spp. are Gram-positive, aerobic, spore-forming rods (1). *Bacillus* species have wide-ranging physiological abilities and are environmentally ubiquitous (2). They are of great importance to soil ecosystems, being involved in diverse functions such as nutrient cycling and plant stress tolerance (3).

Here, we report the draft genomic sequences of Bacillus amyloliquefaciens strain CBMAI 1301 and Bacillus subtilis strain CBMAI 1302, isolated from soybean seeds and soil, respectively. Both strains inhibit common agricultural phytopathogens and have potential applications for their biocontrol, but the underlying genes and metabolic pathways remain unknown. Sequencing of these two genomes could present solutions to relevant issues in soybean cultivation.

CBMAI 1301 was isolated from soybean seeds. The seeds were previously exposed to superficial disinfection in 70% ethanol (1 min), 2.5% sodium hypochlorite (2 min), and 70% ethanol (1 min), followed by two washouts in sterile distilled water. After disinfection, the seeds were ground and agitated in phosphate-buffered saline (PBS) (1 h, 150 rpm), and the suspension containing the bacteria was plated. CBMAI 1302 was isolated from the soil samples by serial dilution. Both strains were incubated (25°C, 96 h) in plates of 10% Trypticase soy agar (TSA; Bacto BD, USA) supplemented with 50 μg/ml cycloheximide and 50 μg/ml nystatin. After incubation, the colonies were purified, kept in TSA, and preserved by lyophilization and ultrafreezing. The samples were reisolated in Luria–Bertani (LB) agar, and genomic DNA was extracted from a single colony cultured with agitation (200 rpm) at 37°C overnight in 10 ml LB medium using a Wizard genomic DNA purification kit (Promega, Madison, WI, USA). The DNA integrity and quality were evaluated by electrophoresis on 1% agarose gel stained with GelRed (Biotium, Inc., Hayward, CA, USA). The DNA purity was assessed using a NanoDrop spectrophotometer (Thermo Fisher Scientific, Inc., Waltham, MA, USA). DNA quantification was performed using a Qubit v4.0 fluorometer (Life Technologies Invitrogen, Carlsbad, CA, USA).

DNA libraries were generated using a Nextera Flex DNA sample preparation kit (Illumina, San Diego, CA, USA). The size distribution of the libraries was evaluated using a 2100 Bioanalyzer (Agilent, Santa Clara, CA, USA). Paired-end sequencing (2 × 150 bp) was performed on an Illumina NextSeq platform at SENAI CETIQT (SENAI Innovation Institute for Biosynthetics, Technology Center and Textile Industry, Rio de Janeiro, RJ, Brazil).

The sequencing of *B. amyloliquefaciens* CBMAI 1301 generated 30,819,703 paired-end reads with an average length of 143.3 bp; the sequencing of B. subtilis CBMAI 1302 generated 15,445,463 paired-end reads with an average length of 143.4 bp. The quality of the raw sequencing data was evaluated using FastQC (4) and preprocessed using Trimmomatic v0.39 (5) with default parameters. The preprocessed reads were *de novo* assembled using SPAdes v3.15 (6) (--isolate -k 21,33,55,77,99,127 --cov-cutoff auto). The assembled partial genome sequence of *B. amyloliquefaciens* CBMAI 1301 comprised 52 scaffolds (longest read, 639,158 bp; *N*_50_, 447,055 bp; estimated genome size, 4,013,009 bp). The GC content of all the assembled scaffolds was 46.2%, and the genome completeness was estimated to be 99.8% (7). The partial genome sequence of B. subtilis CBMAI 1302 comprised 26 scaffolds (longest read, 1,658,102 bp; *N*_50_, 1,040,364 bp; estimated genome size, 4,064,901 bp). The GC content of all the assembled scaffolds was 43.5%, and the genome completeness was estimated to be 99.81%.

The draft genome sequences were annotated using Prokka v1.14.6 (8) with default parameters. In the draft genome sequence of B. subtilis CBMAI 1302, 4,222 protein-coding genes, 14 rRNAs, 84 tRNAs, and 1 transfer-messenger RNA (tmRNA) were annotated. In the draft genome sequence of *B. amyloliquefaciens* CBMAI 1301, 3,930 protein-coding genes, 12 rRNAs, 85 tRNAs, and 1 tmRNA were annotated. The 16S rRNA sequences in both assemblies were assigned to the phylum *Firmicutes* and genus *Bacillus* by IDTAXA (9) (100% confidence) with a classifier trained on the Ribosomal Database Project 16S v18 (10).

### Data availability.

The whole-genome shotgun project has been deposited at DDBJ/ENA/GenBank under the accession number JAFLWZ000000000 for *B. amyloliquefaciens* strain CBMAI 1301 and JAFNJL000000000 for B. subtilis strain CBMAI 1302. The raw reads were deposited in the Sequence Read Archive (SRA) under the accession numbers SRR13920147 and SRR13933388 for CBMAI 1301 and CBMAI 1302, respectively. The genome assembly versions described in this paper are the first versions.
